# Non-Invasive EEG Recordings in Epileptic Dogs (*Canis familiaris*)

**DOI:** 10.3390/vetsci12080758

**Published:** 2025-08-13

**Authors:** Katalin Hermándy-Berencz, Luca Kis, Ferenc Gombos, Anna Paulina, Anna Kis

**Affiliations:** 1 HUNREN Research Centre for Natural Sciences, Institute of Cognitive Neuroscience and Psychology, 1117 Budapest, Hungary; 2 Klapka Animal Health Centre, 2092 Budakeszi, Hungary; 3 HUN-REN-ELTE-PPKE Adolescent Development Research Group, 1088 Budapest, Hungary; 4 Laboratory for Psychological Research, Pázmány Péter Catholic University, 1088 Budapest, Hungary; 5 ELTE-HUNREN NAP Comparative Ethology Research Group, 1117 Budapest, Hungary

**Keywords:** dog, epilepsy, non-invasive EEG, sleep

## Abstract

In addition to characteristic and easily identifiable behavioural signs—namely epileptic seizures—electroencephalography (EEG) has long been a standard component of epilepsy diagnosis protocols. In veterinary practice, EEG is typically performed in a semi-invasive manner, using subcutaneous electrodes and sedation. Here, we propose that the non-invasive polysomnography protocol, originally developed for basic research, can serve as a more welfare-friendly yet informative alternative for assessing epileptic brain activity in dogs. In this study, N = 11 family dogs diagnosed with epilepsy underwent a single non-invasive polysomnography session. EEG-based evidence for epileptic activity was detected in two cases. Polysomnography data from these 11 epileptic dogs were further analysed to evaluate sleep structure parameters. Compared to a matched control group of N = 11 clinically healthy dogs, the epileptic group exhibited reduced sleep efficiency, increased sleep latency, more wakings after sleep onset, and less time spent in drowsiness and non-REM sleep. These findings support the potential utility of non-invasive brain monitoring techniques, such as polysomnography, in the diagnosis and management of epilepsy in veterinary medicine.

## Simple Summary

In this study, researchers tested a non-invasive method called polysomnography to monitor brain activity in pet dogs with epilepsy. This technique, which does not require sedation or surgery, was originally used in research but may also be helpful in veterinary care. Eleven family dogs diagnosed with epilepsy participated in the study. EEG data collected during sleep revealed epileptic activity in two of the dogs. The sleep patterns of epileptic dogs were then compared to those of 11 healthy dogs. The epileptic dogs showed signs of poorer sleep: they took longer to fall asleep, woke up more often during sleep, and spent less time in restful sleep stages. These results suggest that non-invasive brain monitoring during sleep could become a useful and more animal-friendly tool for diagnosing and managing epilepsy in dogs.

## 1. Introduction

Epilepsy is one of the most common chronic neurological disorders globally, affecting both humans and non-human species. The prevalence of epileptic seizures ranges from 1–3% in the human population [1], and 0.5–5.7% in dogs [2,3]. Caring for an epileptic dog requires a substantial and ongoing commitment from the owners, particularly in terms of administering antiepileptic drugs (AEDs) and attending regular veterinary checks [4,5]. In addition to the medical burden, epilepsy also affects the quality of life (QoL) of both affected dogs and their caregivers. Studies have shown that seizure frequency, rather than severity, is associated with lower perceived canine QoL [6]. Dogs on third-line AEDs and those experiencing more severe adverse effects—such as increased sleeping and ataxia—have lower QoL scores [6]. Beyond seizures, epilepsy may also affect dogs’ neurobehavioural, emotional, and cognitive functioning [7]. For caregivers, epilepsy management can be both emotionally and financially taxing, with a median monthly medication cost estimated between $51 and $75 [8]. However, some evidence suggests that dog owners may not perceive the management burden as significantly diminishing QoL for themselves or their pets [9].

While the causes and treatment of epilepsy are well-studied in humans, e.g., [1,10], the disorder remains less well understood in non-human animals. Epilepsy can be classified as idiopathic, structural, and reactive [11]. Numerous parallels have been identified between human and canine epilepsy so far [12]. Structural epilepsy arises from identifiable insults such as trauma, infections, or neurodegeneration. Idiopathic epilepsy, by contrast, is presumed to have a genetic basis and shows high heritability in certain dog breeds—such as Border Collies and Labrador Retrievers [13]. Similarly, human epilepsy often involves a genetic component, though its polygenic nature complicates assessments of heritability [14]. One notable example of genetic convergence is the identification of mutations in the LGI2 gene in both epileptic Lagotto Romagnolo dogs and children with epilepsy [15]. Multidrug-resistant epilepsy also appears in both species [16], and traumatic brain injury is a shared risk factor for post-traumatic epilepsy [17,18].

Electroencephalography (EEG) is a core diagnostic tool for epilepsy in humans, helping determine whether an episode is of epileptic origin and enabling the classification of epilepsy syndromes. In veterinary medicine, however, EEG is underutilized. A recent survey found that fewer than 50% of veterinary neurologists perform EEG, and even among those who do, usage is infrequent [19]. One of the most effective diagnostic contexts for human epilepsy is EEG recording during natural sleep, e.g., [20], but no such data exist for epileptic dogs, as clinical EEGs are typically performed under anaesthesia, e.g., [21], and even pioneering advances to use semi-invasive methods (needle electrode) in order to record natural EEG patterns in dogs (video EEG) have focused on awake recordings [22].

In addition to sleep’s diagnostic value, epilepsy is further associated with sleep–wake cycle disturbances in humans. Patients with focal epilepsy often exhibit reduced rapid eye movement (REM) sleep and decreased sleep efficiency, while those with generalised epilepsy show increased slow-wave sleep and similarly reduced sleep efficiency [23]. Greater social jetlag and irregular sleep–wake patterns across weekdays and weekends have also been reported, along with a correlation between higher seizure frequency and poorer sleep quality [24]. Patients with refractory temporal lobe epilepsy have been found to experience more awakenings after sleep onset compared to both frontal lobe epilepsy patients and healthy controls [25]. Furthermore, epilepsy patients show non-REM sleep instability, characterised by increased cyclic alternating pattern rates and decreased A1 subtype percentages [23]. Although AEDs may partially restore sleep architecture, they do not fully normalise sleep patterns in epilepsy patients [23].

Recent advances in canine neurocognitive research have led to the development of fully non-invasive, welfare-friendly brain monitoring techniques for family dogs [26]. Of particular interest is the canine polysomnography protocol [27], which enables EEG recordings during natural sleep without the need for sedation or prior training. This approach has been successfully used in multiple studies exploring sleep physiology and behaviour in dogs [28], demonstrating its practicality and compatibility with modern animal welfare guidelines.

The present study has two main aims. First, we investigate whether non-invasive sleep EEG, as implemented via canine polysomnography, can detect epileptiform activity in dogs with a clinical diagnosis of epilepsy. Second, we compare the sleep architecture of these epileptic dogs with that of age- and breed-matched healthy controls to identify potential abnormalities in sleep patterns associated with epilepsy.

## 2. Materials and Methods

### 2.1. Patients

Our subjects were N = 11 adult pet dogs (from 2 to 11 years of age, mean: 6.27 years) all diagnosed with epilepsy (4 females, 7 males; all neutered; Table 1). All participants were referred by KHB’s veterinary practice and were receiving antiepileptic medication at the time of the study. Diagnosed seizure types included focal–frontal lobe, generalised clonic, generalised tonic–clonic and mixed forms (e.g., generalised clonic + focal–frontal lobe), with varying intervals between the last seizure and the EEG recording. Magnetic resonance imaging (MRI) scans were available for N = 7 of the dogs.

### 2.2. EEG Measurement

The EEG recordings were conducted using a fully non-invasive and previously validated protocol [27], employing four active electrodes, including bilateral frontal placements (F7, F8) on the right and left zygomatic arch next to the eyes and another two over the anteroposterior midline of the skull (Fz, Cz). All four EEG electrodes were referenced to the G2 electrode, located at the posterior midline (external occipital protuberance). The ground electrode (G1) was placed on the left temporalis muscle. During electrode placement, all dogs were positively reinforced with social interaction (e.g., petting, praise) and/or food rewards. For visualisation purposes, an additional EOG channel was computed as F7–F8 to aid eye movement identification.

EEG signals were collected, pre-filtered, amplified, and digitised at a sampling rate of 1024 Hz per channel using a SAM 25 R MicroMed Headbox (MicroMed Inc., Houston, TX, USA). The hardware passband was set to 0.5–256 Hz, with an anti-aliasing filter cutoff at 1 kHz, and 12-bit resolution across a voltage range of ±2 mV. Additionally, second-order software filters were applied (high-pass > 0.016 Hz, low-pass < 70 Hz) using the System Plus Evolution software (MicroMed Inc., Houston, TX, USA; version 1.05.0001).

### 2.3. Visual Inspection

The recorded EEG traces were visually inspected by a practicing veterinarian (KHB) for any sign of epileptiform activity.

### 2.4. Control Subjects

In order to reveal any potential anomalies in the sleep structure of epileptic dogs, a group (N = 11) of healthy dogs were also measured using the same non-invasive EEG recording protocol. The healthy control group was matched to the patients as much as possible regarding breed, age and gender (Table 2). A further inclusion criterion was that dogs in the control group reported no observed signs of epilepsy or any other neurological condition.

### 2.5. Sleep Macrostructure Scoring

Sleep recordings were visually scored in 20 s epochs according to standardised criteria [27] using a custom-developed software tool (Fercio © Ferenc Gombos, 2012; version 454). This method reliably distinguished between wakefulness, drowsiness, non-REM sleep and REM sleep. Given the variability in the duration of individual recordings the first 40 min (119 epochs) were analysed for all subjects to ensure standardisation. The coded data were then used to extract the following sleep macrostructure variables: sleep efficiency (% of the recording spent in drowsiness, non-REM and REM), sleep latency (time from recording onset to the first occurrence of non-REM sleep), wakings after sleep onset (total time awake after the first drowsiness period; in minutes), drowsiness duration (minutes), non-REM duration (minutes), REM duration (minutes) and REM latency (time from the first drowsiness to the first REM sleep, in minutes).

### 2.6. Statistical Analysis

Epileptic and control dogs were compared on each sleep macrostructure variable using paired t-tests, as the data met the assumption of normality based on the Shapiro–Wilk test. All statistical tests were carried out using JASP software, University of Amsterdam, Amsterdam, The Neatherlands; and IBM SPSS version 22 was used for data visualisation.

## 3. Results

### 3.1. Descriptive Results

**Figure 1:**
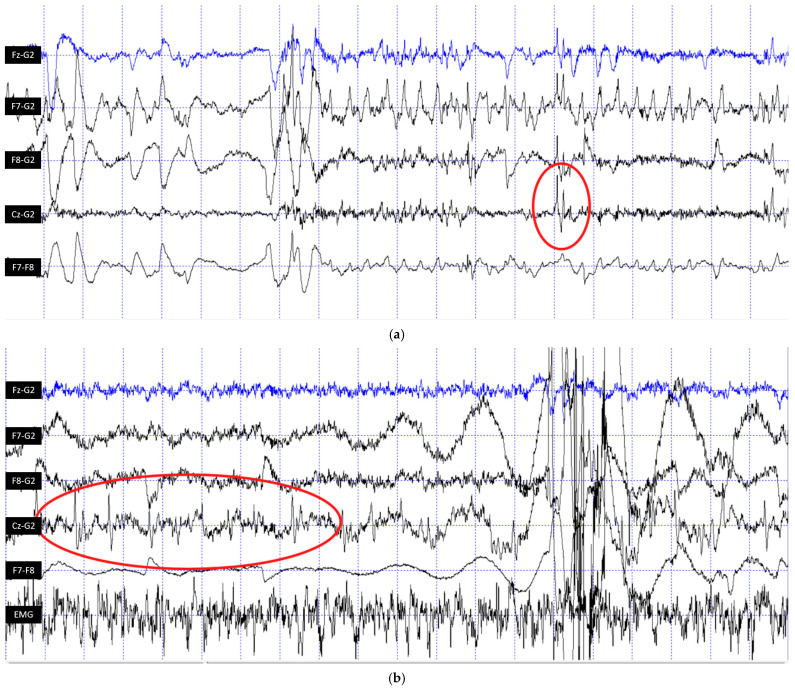
EEG traces of Dog ID 2 (**a**) and Dog ID 3 (**b**) showing the epileptiform activity in a red circle, as per veterinary diagnostic criteria. The EEG derivations from top to bottom are Fz (fronto-central), F7 (right frontal), F8 (left frontal), Cz (central) and EOG (eye movements); the time interval between two dashed lines is 1 s.

Out of the N = 11 patient dogs, N = 3 did not fall asleep during the measurement; thus, their recordings could not be inspected for epileptiform activity due to muscle artefacts associated with awake muscle tone. From the remaining N = 8 dogs, N = 2 provided epilepsy-positive EEG traces (Figure 1), while the EEG recordings of N = 6 dogs were negative.

### 3.2. Sleep Macrostructure Differences

**Figure 2:**
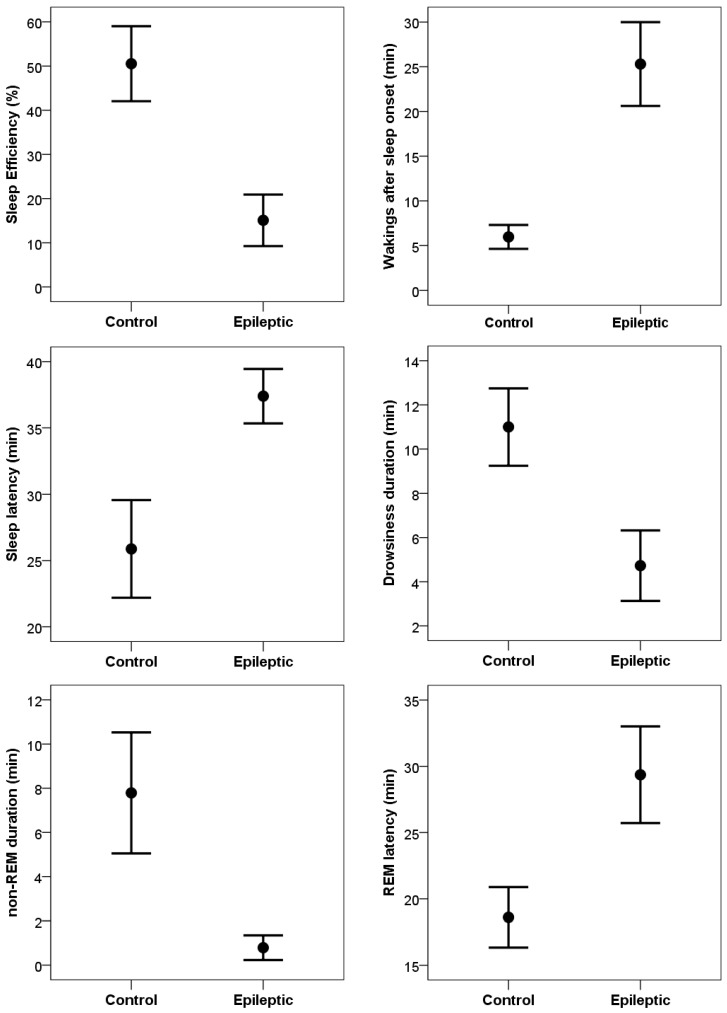
Sleep macrostructure differences (mean ± SE values) between the epileptic patients (N = 11) and matched healthy control dogs (N = 11).

Compared to control subjects, dogs in the epileptic group (Figure 2) had significantly lower sleep efficiency values (t_(10)_ = 3.79, *p* = 0.004; Cohen’s d = 1.14), significantly longer sleep latency (t_(10)_ = 2.45, *p* = 0.035; Cohen’s d = 0.74), and significantly more wakings after sleep onset (t_(10)_ = 4.26, *p* = 0.002; Cohen’s d = 1.29). Epileptic dogs also spent significantly less time in non-REM sleep (t_(10)_ = 3.86, *p* = 0.003; Cohen’s d =1.16) and also less time in drowsiness (t_(10)_ = 3.72, *p* = 0.004; Cohen’s d = 1.12). Time spent in REM sleep did not differ between the epileptic and control groups (t_(10)_ = 1.55, *p* = 0.153; Cohen’s d = 0.47). Latency to reach REM sleep, however, was longer in the epileptic group (t_(10)_ = 2.84, *p* = 0.018; Cohen’s d = 0.86).

## 4. Discussion

The results of the current study demonstrate that epileptiform activity can be detected using EEG traces recorded using the fully non-invasive canine polysomnography protocol. However, some technical challenges were also encountered. A considerable proportion of epileptic dogs did not fall asleep during the single recording session, rendering their data unsuitable for analysis. This finding aligns with previous studies suggesting the need for an adaptation sleep session prior to conducting cognitive experiments [29,30]. It should be noted, however, that previous studies on family dogs have already successfully used single-session recordings (e.g., to quantify the effect of age on sleep spindles [31]) with much lower exclusion rates due to subjects not falling asleep. This, together with the fact that healthy controls in the current study did not have problems falling asleep, suggests that difficulty in sleep onset is characteristic of epileptic dogs, making it challenging to apply the non-invasive polysomnography method for all patients. Alongside existing guidelines indicating that sleep and drowsiness facilitate the activation of epileptiform discharges [32], our results suggest that implementing longer and repeated recordings may be necessary for successful veterinary application of this protocol. Importantly, however, in the case of healthy dogs, there were only minimal differences in the spontaneous sleep pattern of the first versus the second sleeping occasion [29], thus epileptic dogs’ problems falling asleep might extend to repeated recordings as well. A further limitation of the currently used setup is the lack of recording sites over the temporal lobe, which would be necessary to detect temporal lobe epilepsy. There have been some recent attempts to increase the number of recording sites for the non-invasive dog polysomnography protocol [33], which would be a promising future direction to increase diagnostic validity. The use of non-invasive EEG is still advised in its current form due to its welfare-friendly nature compared to needle electrodes, and because epileptiform activity might manifest during natural sleep, which is not detected with traditional measurements under sedation. Based on our current knowledge, the combination of a non-invasive measurement, if negative, followed by a traditional EEG recording [34] might be the best practice in terms of diagnostic validity.

It is also plausible that epileptiform activity is not consistently present in all epileptic dogs during the interval between seizures. Notably, in the current study, epileptiform discharges were observed only in two dogs whose most recent seizures occurred relatively recently (on the previous day and within two weeks), compared to those with negative EEGs where the last seizure occurred up to two months earlier. In human medicine, interictal discharges are detected in only ~50% of known epilepsy cases during the first routine EEG [32], and earlier canine studies using needle electrodes reported abnormal EEG activity in 65% of epileptic dogs [21]. Given these benchmarks, the current detection rate of 25% using a non-invasive protocol is promising. While EEG has long been a standard component of epilepsy diagnosis in human medicine, its veterinary application in dogs has typically relied on invasive methods involving sedation and/or using needle electrodes [35]. This study highlights the potential of non-invasive EEG in the diagnosis of canine epilepsy, and will likely complement modern veterinary practices (such as video EEG and actigraphy) already under testing [36]. Nevertheless, it is important to consider that non-invasive EEG techniques are particularly sensitive to movement, electrode placement accuracy, and individual animal behaviour, all of which can influence data quality and interpretation [37]. The currently experienced underutilisation of EEG in veterinary medicine [19] is likely due to the fact that epileptic dogs are uniformly prescribed the same antiepileptic drugs based on behavioural diagnosis only. However, future research should evaluate if, similarly to human medicine, electroencephalography can aid in the classification of epilepsy syndromes, or have an added prognostic value.

The second key finding of this study concerns the considerable alterations observed in the sleep architecture of epileptic dogs compared to healthy controls. These include decreased sleep efficiency, increased latency to both sleep onset and REM sleep, and reduced time spent in drowsiness and non-REM sleep. It is important to note that all epileptic dogs in the study were undergoing pharmacological treatment. Therefore, based solely on this dataset, it is not possible to disentangle the effects of epilepsy itself from those of the medications, or the possible interaction between the two. Previous studies using activity monitoring suggested that antiepileptic drugs are associated with lethargy and lower baseline activity levels in medicated dogs compared to untreated dogs with idiopathic epilepsy; however, estimated sleep scores did not differ [38]. Similarly, a study involving N = 4 genetically epileptic Beagles found no difference in the percentage of time spent asleep or awake compared to healthy Beagles, although differences in sleep and REM latency were observed [39]—the latter findings are consistent with the present study. In humans, alterations in sleep architecture are known to depend on seizure type [25]. Epileptic dogs in the current study exhibited heterogenic seizure types; however, due to the limited sample size, we could not test whether this has an interacting effect. It is also relevant to consider that antiseizure medications in humans have been shown to partially, but not fully, normalise sleep architecture in epileptic patients [23]. Therefore, the observed sleep abnormalities in the current study are likely attributable, at least in part, to the underlying epileptic condition. Even greater differences might be expected between untreated epileptic dogs and healthy controls.

Beyond the welfare-friendly diagnostic potential of non-invasive EEG in dogs, the observed sleep structure alterations may have further implications for behaviour and cognition due to the deteriorating effect of poor sleep quality [40]. Cognitive decline in epileptic dogs has been shown to be influenced by seizure frequency and the effects of medication [41,42]. Dogs experiencing more frequent seizures exhibit greater cognitive deterioration than those with less frequent episodes. Moreover, memory impairments and disorientation tend to worsen as the disease progresses [42]. The risk of canine cognitive dysfunction is higher among epileptic dogs, particularly in those with a longer history of seizures or with seizure frequency exceeding one episode per week [43]. At present, there is insufficient evidence to guide targeted treatment for the sleep and cognitive disturbances observed in epileptic dogs, and the causal relationship between these symptoms remains unclear. Some interventions have attempted to improve quality of life through physical exercise [43]. While such interventions increased sleep scores as expected [44], they were also associated with a higher monthly seizure frequency [43], highlighting the complexity of epilepsy management.

In conclusion, while the diagnosis and treatment of canine epilepsy remain multifaceted and challenging, non-invasive methods such as canine polysomnography may offer valuable, welfare-conscious tools for improving both diagnosis and monitoring. These tools may ultimately contribute to a better understanding of the interplay between epilepsy, sleep, and cognition in dogs.

## Figures and Tables

**Table 1:** Characteristics of the epileptic dogs participating in the study.

ID	Breed	Age	Gender	Seizure Type	Last Seizure Within	MRI Scan
1	Golden Retriever	2 y	female (n)	focal–frontal lobe	4 months	negative
2	mix	11 y	male (n)	generalised clonic	2 weeks	severe left hemisphere lateral ventriculomegaly
3	Dalmatian	9 y	male (n)	focal–frontal lobe + generalised clonic	1 day	negative
4	Hungarian Vizsla	6 y	male (n)	generalised clonic, with focal–frontal lobe origin	5 months	indication of post-ictal edema or cortical dysplasia
5	Husky	6 y	female (n)	generalised tonic–clonic	2 months	negative
6	mix	6 y	male (n)	focal–frontal lobe + generalised tonic–clonic	3 weeks	negative
7	mix	4 y	female (n)	generalised clonic	3 months	NA
8	French bulldog	10 y	male (n)	generalised tonic–clonic	5 days	NA
9	Pug	6 y	male (n)	generalised tonic–clonic (not verified)	NA	NA
10	Poodle	5 y	female (n)	generalised clonic + focal–frontal lobe	6 weeks	NA
11	Boston Terrier	4 y	male (n)	generalised tonic, with focal–frontal lobe origin	1 week	severe fourth ventricle ventriculomegaly and mild bilateral lateral third ventricle ventriculomegaly

“y” stands for years of age and “(n)” for neutered gender, “NA” for non-applicable.

**Table 2:** Demographic information of patient dogs and matched controls.

ID	Patient Dogs (Epileptic)			Control Dogs (Healthy)		
	Breed	Age	Gender	Breed	Age	Gender
1	Golden Retriever	2 y	female (n)	Golden Retriever	2 y	female (n)
2	mix	11 y	male (n)	mix	11 y	male (n)
3	Dalmatian	9 y	male (n)	Dalmatian	13 y	male (n)
4	Hungarian Vizsla	6 y	male (n)	Hungarian Vizsla	9 y	male (n)
5	Siberian Husky	6 y	female (n)	Siberian Husky	8 y	male (n)
6	mix	6 y	male (n)	mix	5 y	male (n)
7	mix	4 y	female (n)	mix	3 y	female (n)
8	French Bulldog	10 y	male (n)	French Bulldog	10 y	male (n)
9	Pug	6 y	male (n)	Boxer	7 y	male (n)
10	Poodle	5 y	female (n)	Poodle	4 y	female (n)
11	Boston Terrier	4 y	male (n)	Boxer	6 y	male (n)

“y” stand for years of age and “(n)” for neutered gender.

## Data Availability

The raw data supporting the conclusions of this article are stored on the institutional NAS server and will be made available by the authors on request.

## References

[1] Engel J., Timothy A., Pedley A., Aicardi J. (2008). Epilepsy: A Comprehensive Textbook.

[2] Podell M., Fenner W.R., Powers J.D. (1995). Seizure classification in dogs from a nonreferral-based population. J. Am. Vet. Med. Assoc..

[3] Heske L., Nødtvedt A., Jäderlund K.H., Berendt M., Egenvall A. (2014). A cohort study of epilepsy among 665,000 insured dogs: Incidence, mortality and survival after diagnosis. Vet. J..

[4] Lord L.K., Podell M. (1999). Owner perception of the care of long-term phenobarbital-treated epileptic dogs. J. Small Anim. Pract..

[5] Bhatti S.F., De Risio L., Muñana K., Penderis J., Stein V.M., Tipold A., Berendt M., Farquhar R.G., Fischer A., Long S. (2015). International Veterinary Epilepsy Task Force consensus proposal: Medical treatment of canine epilepsy in Europe. BMC Vet. Res..

[6] Wessmann A., Volk H.A., Packer R.M.A., Ortega M., Anderson T.J. (2016). Quality-of-life aspects in idiopathic epilepsy in dogs. Vet. Rec..

[7] Packer R.M.A., Volk H.A. (2015). Epilepsy beyond seizures: A review of the impact of epilepsy and its comorbidities on health-related quality of life in dogs. Vet. Rec..

[8] Nettifee J.A., Munana K.R., Griffith E.H. (2017). Evaluation of the impacts of epilepsy in dogs on their caregivers. J. Am. Anim. Hosp. Assoc..

[9] Masucci M., Di Stefano V., Donato G., Mangano C., De Majo M. (2021). How owners of epileptic dogs living in italy evaluate their quality of life and that of their pet: A survey study. Vet. Sci..

[10] Goldberg E.M., Coulter D.A. (2013). Mechanisms of epileptogenesis: A convergence on neural circuit dysfunction. Nat. Rev. Neurosci..

[11] Shorvon S.D. (2011). The etiologic classification of epilepsy. Epilepsia.

[12] Potschka H., Fischer A., Von Rüden E.L., Hülsmeyer V., Baumgärtner W. (2013). Canine epilepsy as a translational model?. Epilepsia.

[13] Chandler K. (2006). Canine epilepsy: What can we learn from human seizure disorders?. Vet. J..

[14] Löscher W. (2022). Dogs as a Natural Animal Model of Epilepsy. Front. Vet. Sci..

[15] Jokinen T.S., Metsähonkala L., Bergamasco L., Viitmaa R., Syrjä P., Lohi H., Snellman M., Jeserevics J., Cizinauskas S. (2007). Benign familial juvenile epilepsy in lagotto romagnolo dogs. J. Vet. Intern. Med..

[16] Loscher W., Schwartz-Porsche D., Frey H.H., Schmidt D. (1985). Evaluation of epileptic dogs as an animal model of human epilepsy. Arzneim.-Forsch./Drug Res..

[17] Steinmetz S., Tipold A., Löscher W. (2013). Epilepsy after head injury in dogs: A natural model of posttraumatic epilepsy. Epilepsia.

[18] Pitkänen A., Immonen R.J., Gröhn O.H.J., Kharatishvili I. (2009). From traumatic brain injury to posttraumatic epilepsy: What animal models tell us about the process and treatment options. Epilepsia.

[19] Luca J., McCarthy S., Parmentier T., Hazenfratz M., Zur Linden A., Gaitero L., James F.M.K. (2023). Survey of electroencephalography usage and techniques for dogs. Front. Vet. Sci..

[20] Yuan Q., Li F., Zhong H. (2015). Early diagnosis, treatment and prognosis of epilepsy with continuous spikes and waves during slow sleep. Int. J. Clin. Exp. Med..

[21] Berendt M., Høgenhaven H., Flagstad A., Dam M. (1999). Electroencephalography in dogs with epilepsy: Similarities between human and canine findings. Acta Neurol. Scand..

[22] Folkard E., Niel L., Gaitero L., James F.M.K. (2023). Tools and techniques for classifying behaviours in canine epilepsy. Front. Vet. Sci..

[23] Yeh W.C., Lin H.J., Li Y.S., Chien C.F., Wu M.N., Liou L.M., Hsieh C.F., Hsu C.Y. (2022). Rapid eye movement sleep reduction in patients with epilepsy: A systematic review and meta-analysis. Seizure.

[24] Choi S.J., Joo E.Y., Hong S.B. (2016). Sleep-wake pattern, chronotype and seizures in patients with epilepsy. Epilepsy Res..

[25] Sudbrack-Oliveira P., Lima Najar L., Foldvary-Schaefer N., da Mota Gomes M. (2019). Sleep architecture in adults with epilepsy: A systematic review. Sleep Med..

[26] Bunford N., Andics A., Kis A., Miklósi Á., Gácsi M. (2017). *Canis familiaris* as a Model for Non-Invasive Comparative Neuroscience. Trends Neurosci..

[27] Kis A., Szakadát S., Kovács E., Gácsi M., Simor P., Gombos F., Topál J., Miklósi Á., Bódizs R. (2014). Development of a non-invasive polysomnography technique for dogs (*Canis familiaris*). Physiol. Behav..

[28] Bódizs R., Kis A., Gácsi M., Topál J. (2020). Sleep in the dog: Comparative, behavioral and translational relevance. Curr. Opin. Behav. Sci..

[29] Kis A., Szakadát S., Gácsi M., Kovács E., Simor P., Török C., Gombos F., Bódizs R., Topál J. (2017). The interrelated effect of sleep and learning in dogs (*Canis familiaris*); an EEG and behavioural study. Sci. Rep..

[30] Reicher V., Kis A., Simor P., Bódizs R., Gombos F., Gácsi M. (2020). Repeated afternoon sleep recordings indicate first-night-effect-like adaptation process in family dogs. J. Sleep Res..

[31] Iotchev I.B., Kis A., Turcsán B., Tejeda Fernández de Lara D.R., Reicher V., Kubinyi E. (2019). Age-related differences and sexual dimorphism in canine sleep spindles. Sci. Rep..

[32] De Risio L., Platt S. (2014). Canine and Feline Epilepsy: Diagnosis and Management.

[33] Mondino A.A., Gutiérrez M.A., González C.A., Mateos D.C., Torterolo P.E., Delucchi L.A., Fe S. (2022). Electroencephalographic Signatures of Canine Cognitive Dysfunction. bioRxiv.

[34] Ákos P., Thalhammer J., Leschnik M., Halász P. (2012). Electroencephalographic examination of epileptic dogs under propofol restraint. Acta Vet. Hung..

[35] Jeserevics J., Viitmaa R., Cizinauskas S., Sainio K., Jokinen T.S., Snellman M., Bellino C., Bergamasco L. (2007). Electroencephalography Findings in Healthy and Finnish Spitz Dogs with Epilepsy: Visual and Background Quantitative Analysis. J. Vet. Intern. Med..

[36] Folkard E., McKenna C., Monteith G., Niel L., Gaitero L., James F.M.K. (2023). Feasibility of in-home electroencephalographic and actigraphy recordings in dogs. Front. Vet. Sci..

[37] Kulgod A., van der Linden D., França L.G.S., Jackson M., Zamansky A. (2025). Non-invasive canine electroencephalography (EEG): A systematic review. BMC Vet Res..

[38] Barry M., Cameron S., Kent S., Barnes-Heller H., Grady K. (2021). Daytime and nocturnal activity in treated dogs with idiopathic epilepsy compared to matched unaffected controls. J. Vet. Intern. Med..

[39] Wauquier A., Van Den Broeck W.A.E., Edmonds H.L. (1986). Sleep in epileptic beagles and antiepileptics. Funct. Neurol..

[40] Bolló H., Kovács K., Lefter R., Gombos F., Kubinyi E., Topál J., Kis A. (2020). REM versus Non-REM sleep disturbance specifically affects inter-specific emotion processing in family dogs (*Canis familiaris*). Sci. Rep..

[41] Packer R.M.A., McGreevy P.D., Salvin H.E., Valenzuela M.J., Chaplin C.M., Volk H.A. (2018). Cognitive dysfunction in naturally occurring canine idiopathic epilepsy. PLoS ONE.

[42] Winter J., Packer R.M.A., Volk H.A. (2018). Preliminary assessment of cognitive impairments in canine idiopathic epilepsy. Vet. Rec..

[43] Grady K., Cameron S., Kent S.P., Barnes Heller H., Barry M.M. (2023). Effect of an intervention of exercise on sleep and seizure frequency in idiopathic epileptic dogs. J. Small Anim. Pract..

[44] Bunford N., Reicher V., Kis A., Ákos P., Ferenc G., Bódizs R., Gácsi M., Pogány Á., Gombos F., Bódizs R. (2018). Differences in pre-sleep activity and sleep location are associated with variability in daytime/nighttime sleep electrophysiology in the domestic dog. Sci. Rep..

